# Ventricular Fibrosis and Coronary Remodeling Following Short-Term Exposure of Healthy and Malnourished Mice to Bisphenol A

**DOI:** 10.3389/fphys.2021.638506

**Published:** 2021-04-12

**Authors:** Marta García-Arévalo, Estela Lorza-Gil, Leandro Cardoso, Thiago Martins Batista, Thiago Reis Araujo, Luiz Alberto Ferreira Ramos, Miguel Arcanjo Areas, Angel Nadal, Everardo Magalhães Carneiro, Ana Paula Davel

**Affiliations:** ^1^Department of Structural and Functional Biology, Institute of Biology, Campinas, Brazil; ^2^Obesity and Comorbidities Research Center-OCRC, UNICAMP, Campinas, Brazil; ^3^Instituto de Biología Molecular y Celular, Instituto de Investigación, Desarrollo e Innovación en Biotecnología Sanitaria de Elche, Spanish Biomedical Research Center in Diabetes and Associated Metabolic Disorders, Universidad Miguel Hernández, Elche, Spain

**Keywords:** bisphenol-A, low-protein diet, blood pressure, *Myocardial fibrosis*, coronary vessels

## Abstract

Bisphenol-A (BPA) is an endocrine disruptor associated with higher risk of insulin resistance, type 2 diabetes, and cardiovascular diseases especially in susceptible populations. Because malnutrition is a nutritional disorder associated with high cardiovascular risk, we sought to compare the effects of short-term BPA exposure on cardiovascular parameters of healthy and protein-malnourished mice. Postweaned male mice were fed a normo- (control) or low-protein (LP) diet for 8 weeks and then exposed or not to BPA (50 μg kg^−1^ day^−1^) for the last 9 days. Systolic blood pressure was higher in BPA or LP groups compared with the control group. However, diastolic blood pressure was enhanced by BPA only in malnourished mice. Left ventricle (LV) end diastolic pressure (EDP), collagen deposition, and CTGF mRNA expression were higher in the control or malnourished mice exposed to BPA than in the respective nonexposed groups. Nevertheless, mice fed LP diet exposed to BPA exhibited higher angiotensinogen and cardiac TGF-β1 mRNA expression than mice treated with LP or BPA alone. Wall:lumen ratio and cross-sectional area of intramyocardial arteries were higher either in the LP or BPA group compared with the control mice. Taken together, our data suggest that short-term BPA exposure results in LV diastolic dysfunction and fibrosis, and intramyocardial arteries inward remodeling, besides potentiate protein malnutrition-induced hypertension and cardiovascular risk.

## Introduction

Bisphenol-A (BPA) is one of the highest-production-volume chemical used worldwide (Vandenberg et al., 2009). It is used to produce polycarbonate plastic and epoxy resin, which are found in lining food and beverage cans as well as drinking water bottles and containers (vom Saal et al., 2007). BPA is toxic and known to be an endocrine disruptor chemical (EDC). The World Health Organization (WHO) defines EDCs as exogenous agents or a mix thereof that alters endocrine system functions and consequently causes adverse effects on intact organisms, their progeny, or (sub)populations (Damstra et al., 2002).

The first endocrine disruptor statement of the Endocrine Society reviewed evidence related to EDC exposure and the increased risk of cardiovascular disease (CVD) (Diamanti-Kandarakis et al., 2009). The document proposed a link between exposure to BPA and increased incidence of CVD. Initially, this effect was ascribed to the capacity of EDCs to act as obesogens. More recently, studies have suggested that EDCs may also increase the cardiovascular risk by a direct effect on the cardiovascular system (Gore et al., 2015). In accordance, data from the National Health and Nutrition Examination Survey (NHANES) showed a significant association between high urinary BPA concentration and the occurrence of coronary artery disease, even when adjusted for traditional risk factors as body mass index (Melzer et al., 2010, 2012b). Adverse effects of BPA seem to be dependent on dose, gender, exposure time, and coexposure with other risk factors (Wehbe et al., 2020; Zhang et al., 2020). In addition, BPA exposure has been associated with CVD such as peripheral arterial diseases, myocardial infarction, arrhythmias, dilated cardiomyopathy, atherosclerosis, and hypertension (Shankar and Teppala, 2012; Zhang et al., 2020). Despite these epidemiological data, the mechanisms underlying susceptibility to CVD in BPA-exposed individual remain unclear.

Epidemiological studies (Lang et al., 2008; Shankar and Teppala, 2011; Silver et al., 2011; Wang et al., 2012) and animal models (Alonso-Magdalena et al., 2010, 2011; Angle et al., 2013; Liu et al., 2013; Garcia-Arevalo et al., 2014, 2016) have shown that exposure to BPA enhances the risk of metabolic disorders which may contribute to accelerate the onset of and/or aggravate pre-existing CVD. Interestingly, tolerable daily intake of 50 μg kg^−1^ day^−1^ of BPA can increase circulating inflammatory factors in mice under low-caloric diet but not on high-fat diet (Yang et al., 2016). Caloric and protein restriction at early stages of development may induce metabolic and cardiovascular alteration in adult life. Protein restriction during fetal or postweaning development increases blood pressure and induces vascular remodeling (Franco Mdo et al., 2002; Brawley et al., 2003; Torrens et al., 2006, 2009; Franco et al., 2008; Maia et al., 2014). In addition, a postweaning low-protein (LP) diet is associated with impaired cardiomyocytes contractility and myocardial fibrosis (Penitente et al., 2013). These phenomena are associated with disrupted redox homeostasis, increased sympathetic tone, and renin-angiotensin system activity (Oliveira et al., 2004; Loss Ide et al., 2007; Maia et al., 2014). Therefore, these mechanisms associated with malnutrition could turn individuals susceptible to cardiovascular damage induced by additional risk factors.

Because EDCs have been suggested to increase CVD incidence, especially in susceptible populations, in the present study, we sought to investigate the effect of short-term exposure to low doses of BPA on mice exhibiting cardiovascular risk due to postweaning protein malnutrition compared with healthy mice. We evaluated the effects of BPA exposure and the combination of BPA and LP diet on blood pressure, left ventricular hemodynamics and morphology, and on the expression of renin-angiotensin system genes.

## Methods

### Drugs

Bisphenol-A [4,4′-isopropylidenediphenol (BPA)] (cat no. 155118) and tocopherol-stripped corn oil (cat no. 901415) were obtained from MP Biomedicals (Solo, OH, USA). BPA was dissolved in tocopherol-stripped corn oil. Animals were treated by a subcutaneous injection with 25 μg BPA kg^−1^ body weight in 100 μl of oil twice a day (8:00 and 20:00). The cumulative dose per day was 50 μg kg^−1^ day^−1^. This dose is in accordance with the safety limit recommended by the Environmental Protection Agency of the USA. Xylazine and ketamine were purchased from Ceva (Paulínia, SP, Brazil).

### Experimental Animals

All experimental protocols were approved by the ethics committee at UNICAMP (protocol no. 3638-1) and conformed to the guidelines for ethical conduct in the care and use of animals established by the National Board of Animal Experimentation Control (CONCEA). Male Swiss mice were purchased from the Multidisciplinary Center for Biological Research at UNICAMP (Campinas, SP, Brazil).

Weaned mice (21 days old) were fed for 8 weeks with a chow diet containing a normal protein level (14% protein) or with an isocaloric low-protein (LP) diet (6% protein). Diet compositions (Pragsoluções Biociências, Jaú—SP, Brazil) were previously described (Batista et al., 2013). On the last 9 days of diet feeding, mice were randomly separated to be exposed to BPA (50 μg kg^−1^ day^−1^) or vehicle (tocopherol-stripped corn oil), resulting in four experimental groups: control (chow diet + vehicle); BPA (chow diet + BPA exposure); LP (LP diet + vehicle); and LPBPA (LP diet + BPA exposure). BPA dose is the safety limit recommended by the Environmental Protection Agency of the USA. During the protocol, mice were maintained at 22 ± 1°C on a 12-h light/dark cycle with free access to water and food.

### Arterial and Left Ventricular Pressure Measurements

Mice were anesthetized with xylazine/ketamine (100 and 10 mg kg^−1^ body weight, respectively) and then catheterized through the right carotid artery for arterial blood pressure and heart rate recording using a pressure transducer (MTL844 AdInstruments, Sydney-NSW, Australia) at a sample rate of 1 kHz (LabChart 7, AdInstruments). Subsequently, the catheter was advanced retrograde across the aortic valve into the left ventricle under continuous hemodynamic monitoring to ensure proper placement in the left ventricle. After stabilization, systolic and end diastolic left ventricular (LV) pressure, d*P*/d*t*_max_ (maximum rate of LV pressure rise) and d*P*/d*t*_min_ (rate of LV pressure fall) were assessed. After the hemodynamic measurements, the mice were euthanized. Then, the heart and lungs were removed en bloc, followed by liver and kidney isolation. Next, organs were weighed and processed as described below.

### LV and Intramyocardial Arteries Histological Analysis

The heart was fixed in 4% paraformaldehyde for 24 h at 4°C. The left ventricle was isolated and embedded in paraffin, and 5-μm-thick sections were obtained. Histological sections were stained with hematoxylin and eosin (H&E) for cardiomyocyte diameter and coronary vessel morphometry and with Masson's trichrome for collagen. Images were acquired at ×40 magnification with a camera (Olympus DP72; software Image-Pro 6.3) connected to a microscope (Olympus BX51) and analyzed by using Image J software.

Cardiomyocyte diameter was measured from 160 to 200 cells/animal and cross-sectional area calculated (*CSA* = πdiameter^2^/4). Suitable cross-sections were defined as having a visible nucleus. Interstitial collagen was quantified from four to five fields taken randomly for each animal avoiding perivascular areas. Collagen deposition was expressed as the percentage of the evaluated LV area.

Intramyocardial arteries internal and external perimeters were measured in LV transversal sections. To decrease the experimental error, each perimeter was taken in triplicate from at least five vessels per animal. Lumen diameter (π internal perimeter), wall thickness (WT = (external diameter−internal diameter)/2), wall/lumen ratio (WT/lumen diameter), and wall CSA (π external diameter^2^− *internal diameter*^2^/4) were calculated.

### Plasma Levels of Catecholamines

Blood samples were collected with EDTA (1 mM) and sodium metabisulfite (4 mM) to prevent catecholamine degradation. Noradrenaline and adrenaline were measured by ELISA kits (LDN immunoassays BA E5200 and BA E-5100, respectively) following the user's handbook.

### Gene Expression by Real-Time Quantitative PCR

Quantitative PCR assays were performed using 7500 Real-Time PCR System (Applied Biosystems, Foster City, CA). Heart (left ventricle) and kidney mRNA was extracted was made with Trizol Reagent (Ambion), and 1 μg of RNA was used for retrotranscription reaction (HighCapacity cDNA Reverse transcription, Applied Biosystems). Expression levels were normalized to the expression of ribosomal protein large P0 gene (*Rplp0*; also known as *36B4)* and *Hrpt*. The resulting values were expressed as the relative expression respect to control levels (2^−Δ*ΔCT*^) (Kubista et al., 2006). Primer sequences are listed in the Supplementary Table 1.

### Kidney Morphology

After euthanasia, the kidneys were immediately perfused with PBS (0.15 M NaCl containing 10 mM sodium phosphate buffer, pH 7.4) at 20 ml min^−1^ through the distal aorta using a peristaltic perfusion pump (Milan Scientific Equipment, BP 600 PR, Brazil). The left kidney was isolated, removed, weighed, and cut in three sections. The medial region of the left kidney was fixed in 10% buffered formalin, dehydrated, and embedded in paraffin for morphological studies. For these assays, 4-μm-thick kidney sections were stained using periodic acid–Schiff (PAS) method and renal morphology was evaluated blindly by one independent person by using a light microscope (Eclipse 80i, Nikon). For the planar glomerular area analysis, all glomeruli with apparent macula dense and afferent arterioles from the renal cortical area of each mouse were included. The area of each glomerulus was determined, and the mean glomeruli areas were obtained using morphometry software (NIS-Elements D, Nikon, Tokyo, Japan).

### Immunofluorescence

Kidney sections (4 μm thick) were deparaffinized, and nonspecific protein binding was blocked by incubation with 3% BSA in PBS for 60 min. Then, kidney sections were incubated overnight at 4°C with a specific primary antibody against α-smooth muscle actin (α-SMA) rabbit polyclonal antibody (1:200; Abcam, Cambridge, MA), and subsequently with an anti-rabbit secondary antibody Alexa fluor 568 (1:100) for 2 h at room temperature. Then, the kidney slices were washed three times with PBS and then stained with 4′6-diamidine-2′-phenylindole dihydrochloride (DAPI; Sigma Aldrich) for 5 min at room temperature. The reaction products were washed three times with PBS and then incubated with 10 mM copper sulfate (CUSO_4_) at room temperature for 10 min, followed by two washes with PBS. The kidney slices were mounted with Dako fluorescent mounting medium (Dako North American Inc. CA, USA) and analyzed using a computerized morphometry program (NIS-Elements, Nikon), whose microscope is equipped with a ×20 objective, a laser excitation of 543 nm to Alexa fluor 568 acquisition and 405 nm to DAPI acquisition.

### Data Analysis

The results are expressed as the mean ± SEM. Normality was tested by Shapiro-Wilk test. Data were analyzed by one-way ANOVA followed by Newman-Keuls multiple comparison using GraphPad Prism 6.0 software (GraphPad Software In, San Diego, CA, USA). Values of *p* < 0.05 were considered significantly different.

## Results

### Short-Term BPA Exposure Did Not Affect Body Weight and Relative Organ Weight

Mice fed an isocaloric LP diet for 8 weeks exhibited lower body weight and heart weight compared with mice fed a chow diet, although heart weight to body weight ratio did not differ (Supplementary Table 2). Exposure to BPA for 9 days also did not affect body weight, heart mass, and the heart mass to body weight ratio in control mice or mice fed a LP diet. Besides, neither BPA nor LP diet affected kidney, lung, and liver weight as well as the percentage of water retention in lungs and liver were similar in the four groups (Supplementary Table 2).

### BPA Exposure in the Malnutrition Background Increased Diastolic Blood Pressure

**Figure 1:**
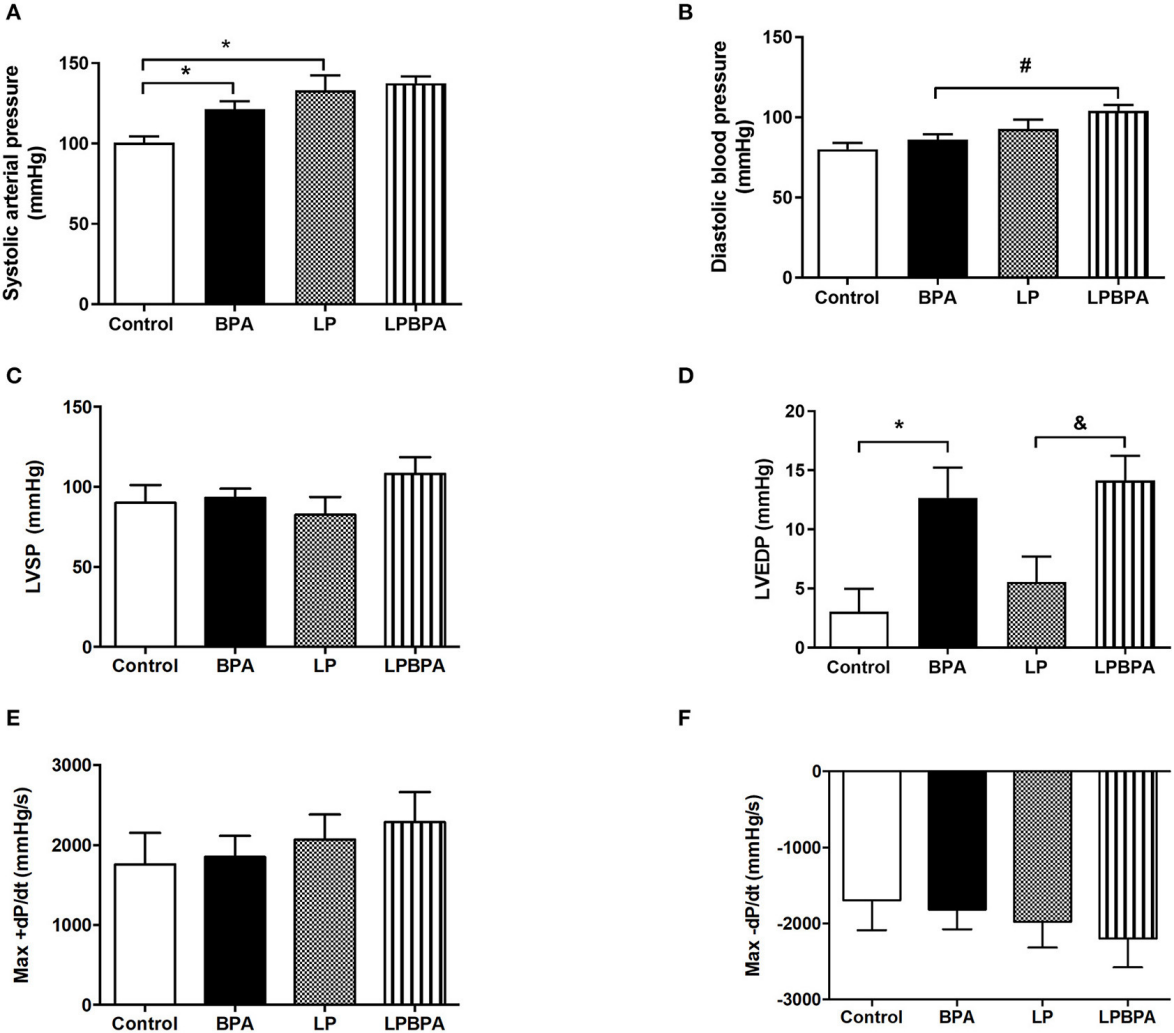
Effects of bisphenol A (BPA) exposure and low protein (LP) diet in hemodynamic parameters. Systolic **(A)** and diastolic **(B)** blood pressure, left ventricular (LV) systolic pressure (LVSP) **(C)**, end diastolic pressure (LVEDP) **(D)**, and maximum positive (Max+) **(E)** and negative (Max-) **(F)** pressure derivatives (d*P*/d*t*) in mice fed normoprotein (control) or LP diet during 8 weeks and exposed to BPA for 9 days. Data are expressed as the mean ± SEM (number of animals/group: Control = 8; BPA = 5; LP = 5; LPBPA = 5). One-way ANOVA followed by the Newman-Keuls test, *p < 0.05 vs. control; ^&^*p* < 0.05 vs. LP; ^#^*p* < 0.05 vs. BPA.

LP or BPA-exposed mice exhibited higher systolic blood pressure with no changes on diastolic blood pressure compared with the control group (Figures 1A,B). Nevertheless, diastolic blood pressure was higher only in LPBPA mice (Figures 1A,B). During blood pressure measurement, heart rate was registered in anesthetized animals and was higher in LPBPA compared with the BPA group (bpm: control= 201 ± 15; BPA = 214 ± 15; LP = 240 ± 38; LPBPA = 328±39^#^; ^#^*p* < 0.05 vs. BPA).

The plasma levels of catecholamines were not statistically different in the four groups but, noteworthy is the three times increase in the concentration of circulating adrenaline in BPA-exposed vs. nonexposed groups (Supplementary Table 2).

### Short-Term BPA Exposure Increases LV End Diastolic Pressure, Collagen Deposition, and Gene Expression of Profibrotic Factors in Control and Malnourished Mice

**Figure 2:**
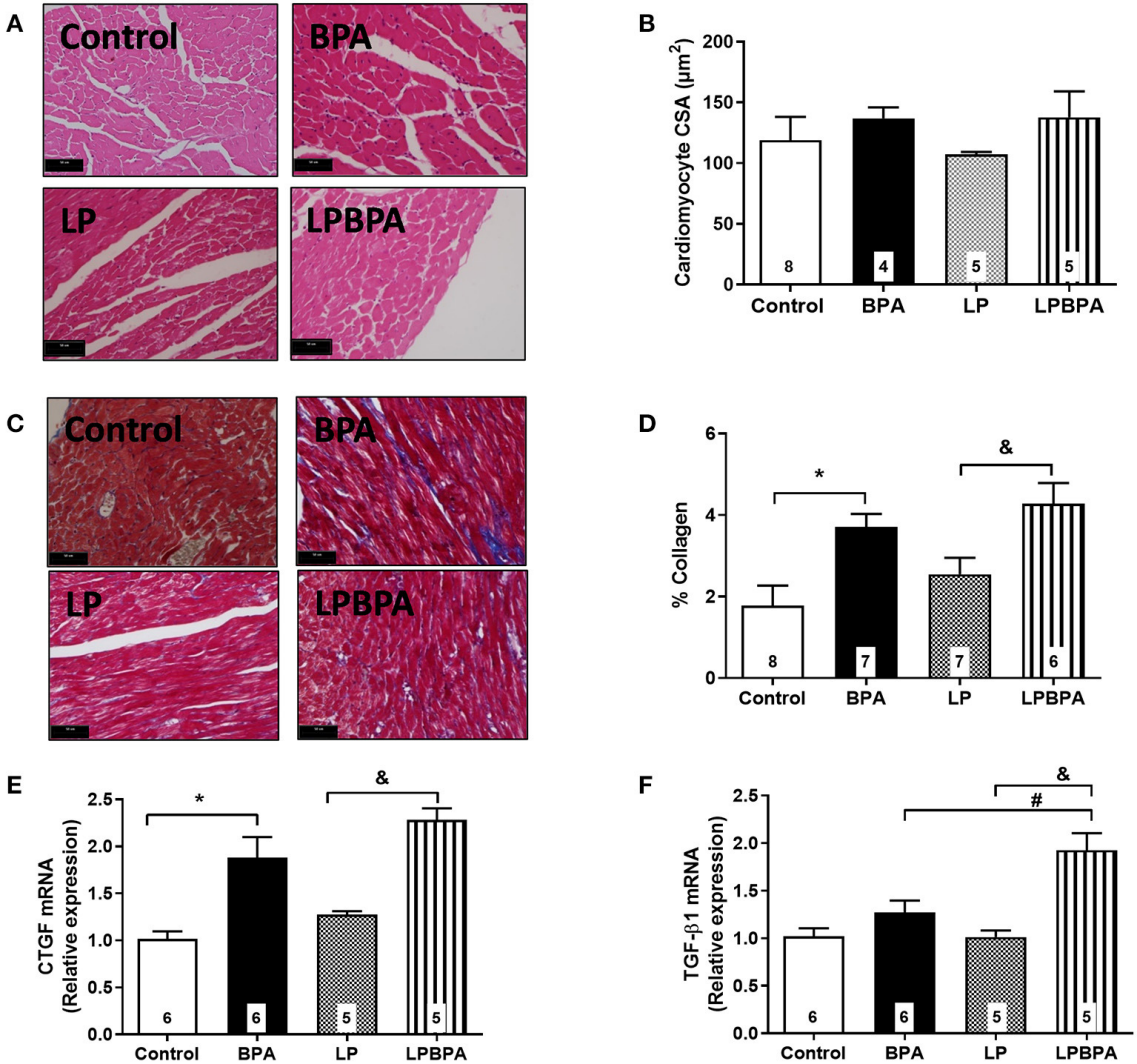
Cardiomyocyte area, collagen deposition, and profibrotic gene expression in control and malnourished mice exposed to BPA. Representative images (bar = 50 μm; ×40 magnification) for H.E. **(A)** and Masson staining **(C)**, cardiomyocyte cross-sectional area (CSA, **B**), and % collagen deposition **(D)** in the myocardial left ventricle of mice fed normoprotein (control) or low-protein (LP) diet during 8 weeks and exposed to BPA for 9 days. CTGF **(E)** and TGF-β1 **(F)** mRNA expression was quantified in LV samples. Data are expressed as the mean ± SEM (number of animals/group is indicated in the bars). One-way ANOVA followed by the Newman-Keuls test; *p < 0.05 vs. control; ^&^*p* < 0.05 vs. LP; ^#^*p* < 0.05 vs. BPA.

Neither protein restriction nor BPA exposure affected LV systolic pressure (Figure 1C) or positive and negative d*P*/d*t* (Figures 1E,F). However, BPA exposure by itself or in combination with LP diet enhanced LV end diastolic pressure (LVEDP) as compared with non-BPA-exposed groups (Figure 1D). In addition, LV cardiomyocyte diameter (data not shown) and CSA (Figures 2A,B) were not significantly affected either by LP diet or BPA.

Interstitial collagen deposition was not significantly changed by LP diet, but it was exacerbated (2.5×) by BPA in both control and LP mice (Figures 2C,D). mRNA expression of connective tissue growth factor (CTGF) and TGF-β1 was not modified by LP diet alone (Figures 2E,F). BPA exposure increased CTGF mRNA expression when compared to non-exposed control and LP mice (Figure 2E), while TGF-β1 mRNA expression was increased by BPA in malnourished mice only (Figure 2F).

### Remodeling of Intramyocardial Arteries Following BPA Exposure or Postweaning Protein Malnourishment

**Figure 3:**
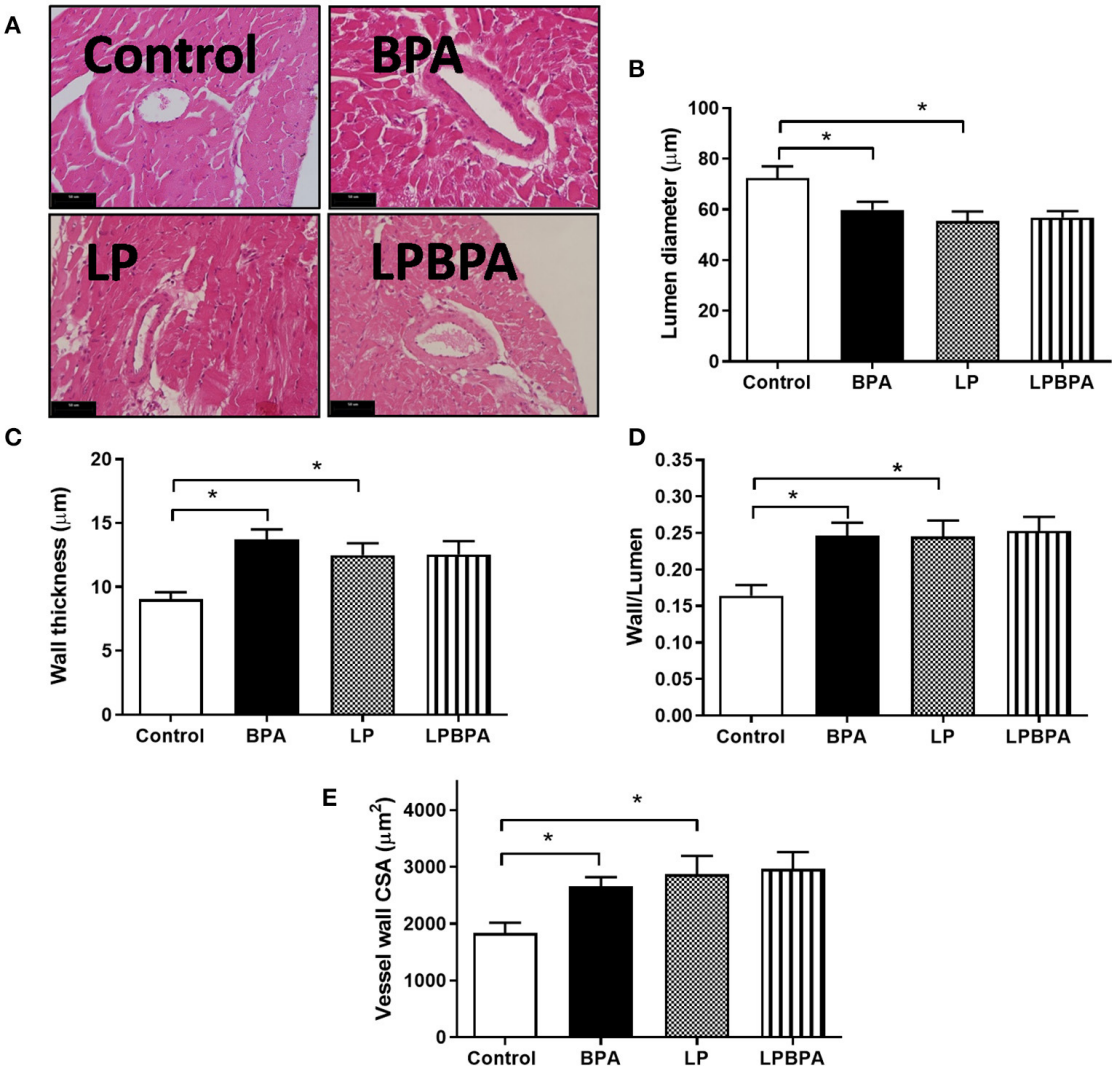
Intramyocardial arterial remodeling following bisphenol A (BPA) and low-protein (LP) diet. Representative images for H.E. (**A**, bars = 50 μm) and quantified lumen diameter **(B)**, wall thickness **(C)**, wall/lumen ratio **(D)**, and wall cross-sectional area (CSA, **E**) of intramyocardial coronary vessels in mice fed normoprotein (control) or LP diet during 8 weeks and exposed to BPA for 9 days. Data are expressed as the mean ± SEM (number of animals/group: control = 10; BPA = 9; LP = 6; LPBPA = 6). One-way ANOVA followed by the Newman-Keuls test; *p < 0.05 vs. control.

Figures 3A–E show that LP diet as well as BPA exposure resulted in intramyocardial arteries with reduced lumen diameter, increased wall thickness, increased wall/lumen ratio, and increased wall CSA, compared with the control group. The combination of BPA and LP diet did not induce additional changes in these morphometrical parameters (Figures 3A–E). Taken together, these results suggest an inward remodeling of intramyocardial arteries of mice exposed to either BPA or LP diet.

### Kidney Morphology Is Not Affected Either by LP Diet or BPA Exposure

Neither BPA exposure nor LP diet changed the glomerular area (Supplementary Figures 1A,B). In addition, no changes were found on α-SMA expression, a marker for vascular smooth muscle cells and myofibroblasts (Supplementary Figures 1C,D). Moreover, no significant changes were detected on protein excretion (data not show). LP diet did not modify renal mRNA expression of CTGF and TGF-β1 while BPA-exposed mice showed increased CTGF compared with non-exposed control and LP mice (Supplementary Figures 1E,F). TGF-β1 mRNA expression was increased by BPA in malnourished mice only (Supplementary Figures 1E,F).

### Renin-Angiotensin System Gene Expression Profile After LP Diet and BPA Exposure

The cardiac and renal gene expression of angiotensin II receptor type 1a (*Agtr 1a*), type 1b *(Agtr1b*), and type 2 (*Agtr2*) were not affected by LP diet or BPA exposure (Table 1). However, the hepatic gene expression of the angiotensin II precursor angiotensinogen (*Agt)* was significantly increased in LPBPA group compared with BPA and LP groups (Table 1).

**Table 1:** Renin-angiotensin system gene expression in mice following postweaning low-protein diet (LP) and/or bisphenol A (BPA) exposure.

	**Control**	**BPA**	**LP**	**LPBPA**
**Heart**
*Agtr1a*	1 ± 0.16	1.78 ± 0.41	1.06 ± 0.14	1.13 ± 0.23
*Agtr1b*	1 ± 0.29	0.88 ± 0.22	0.73 ± 0.21	0.98 ± 0.44
*Agtr2*	1 ± 0.13	1.28 ± 0.36	1.23 ± 0.25	2.16 ± 0.53
**Kidney**
*Agtr1a*	1 ± 0.13	1.02 ± 0.07	1.09 ± 0.16	1.32 ± 0.19
*Agtr1b*	1 ± 0.08	1.20 ± 0.19	1.65 ± 0.29	1.43 ± 0.64
*Agtr2*	1 ± 0.10	1.51 ± 0.17	1.79 ± 0.12	1.96 ± 0.64
**Liver**
*Agt*	1 ± 0.26	0.62 ± 0.10	1.10 ± 0.28	2.36 ± 0.67^&^^,^ ^#^

*Data are mean ± SEM (n = 4–6 animals/group). One-way ANOVA followed by the Newman-Keuls test*;

& *p < 0.05 vs. LP*;

# *p < 0.05 vs. BPA*.

## Discussion

There are several epidemiological studies that show a correlation between urine BPA concentration and the risk for CVD (Lang et al., 2008; Melzer et al., 2010; Shankar and Teppala, 2012). High levels of BPA has been independently associated with higher blood pressure and coronary heart disease (Bae et al., 2012, 2016; Bae and Hong, 2015). In the present study, we demonstrated that short-term exposure to low BPA concentrations might predispose individuals to cardiac diastolic dysfunction and fibrosis and coronary vessels narrowing. In addition, BPA exposure further increases protein malnutrition-induced blood pressure elevation associated with an upregulation of angiotensinogen gene expression. The data suggest that short-term exposure to BPA can induce cardiovascular injury and exacerbate existent cardiovascular complications in a susceptible malnourished population. WHO data revealed that 462 million people are underweight, and 144 million children are stunted. Deaths among children linked to undernutrition mostly occur in low- and middle-income countries that also have weak or non-existent environmental policies to control toxic exposure.

BPA exposure and undernutrition are both risk factors for cardiovascular complications. It is known that protein restriction, either *in utero* or after weaning may produce a slight but significant increase in blood pressure (Franco Mdo et al., 2002; Oliveira et al., 2004; Torrens et al., 2006, 2009; Loss Ide et al., 2007; Maia et al., 2014). In addition, BPA exposure exerts adverse effect on blood pressure (Bae and Hong, 2015; Bae et al., 2016). Our findings confirm these previous reports, as either a postweaning protein-restricted diet or daily exposure to BPA at a dose that is considered safe by the US Environmental Protection Agency, increased systolic blood pressure in mice. To investigate a possible additive adverse effect of BPA exposure in undernourishment, we exposed protein-restricted mice to BPA. We observed that the hypertensive effect of BPA was even greater in this sensitive population, in which BPA administration enhanced diastolic blood pressure. These data along with BPA-associated adverse cardiovascular outcomes particularly in sensitive populations, including fetal, infant, and pediatric groups (Ramadan et al., 2020).

It has been suggested that BPA impact on heart health become even more evident following an adverse cardiovascular event (Patel et al., 2015). Therefore, in the present study, we sought to investigate potential harmful effects of BPA exposure on LV structure and function of mice fed a LP diet, as this is a cardiovascular risk factor particularly at the early stages of development. The postweaning LP diet alone reduced heart mass in mice, although heart weight to body weight ratio was not affected, as previously demonstrated (Murca et al., 2012). In addition, LP diet per se did not significantly change basal LV hemodynamics, cardiomyocyte diameter, or collagen deposition. There are controversial data about the effects of a LP diet on LV hemodynamic, morphology, and contractility. Postweaning LP diet was previously associated with LV hypertrophy and increased cardiac contractility and +d*P*/d*t* and –d*P*/d*t* values in adult life (Murca et al., 2012; de Belchior et al., 2016). In contrast, impaired cardiomyocyte contractility, lower calcium amplitude, reduced cardiomyocyte size, and increased number of collagen fibers in the LV myocardium in response to a postweaning LP diet was reported (Penitente et al., 2013, 2014). Such differences may be related to the stage of the hypertensive disease induced by postweaning protein restriction (Mendes et al., 2017) as well as to differences in LP diet composition. A limitation of our study is that the hemodynamic LV measurements were performed in anesthetized animals.

Short-term BPA exposure in healthy or protein-restricted mice did not impact on body weight, heart mass, LVSP, or LV cardiomyocytes CSA. However, there was an increase in interstitial myocardial collagen content and LVEDP in animals exposed to BPA alone or combined with LP diet, suggesting that fibrosis is implicated in the reduction of diastolic function following BPA exposure, in agreement with the profibrotic effect reported for low doses of BPA (Belcher et al., 2015). A limitation of our study is that Masson trichrome stain other extracellular matrix proteins in addition to collagen. Hu et al. (2016) demonstrated that the increased myocardial collagen content in mice exposed to BPA is associated with MAPK ERK1/2 signaling pathway activation and with increased expression of the profibrotic factor TGF-β1. Also, prenatal exposure to BPA leads to fibrosis in fetal heart that was not associated with hepatotoxicity (Rasdi et al., 2020). Our study showed that short-term BPA exposure enhances the gene expression of CTGF while TGF-β1 was upregulated in the malnourished background only. Taken together, the present data revealed a potential harmful consequence from exposure to a dose of BPA that is predicted to be safe. This can be particularly harmful in a prehypertensive malnourished population.

An association between urinary BPA concentration and coronary artery stenosis has been reported (Melzer et al., 2012a,b). Our findings support a causal role for BPA exposure in coronary arterial remodeling, as *in vivo* exposure to BPA results in reduced lumen diameter and increased wall/lumen ratio and vessel wall CSA of mouse intramyocardial arteries. Nanomolar BPA was recently demonstrated to stimulate the proliferation of vascular smooth muscle cells *in vitro* (Gao et al., 2019). In addition, a longer exposure to BPA and at higher doses may exacerbate atherosclerotic lesions by increasing smooth muscle cell-positive areas and coronary stenosis in hyperlipidemic rabbits (Fang et al., 2014, 2015). Endoplasmic reticulum stress and the expression of inflammatory factors in endothelial cells were suggested as underlying mechanisms involved in BPA-induced vascular damage (Fang et al., 2014, 2015). Therefore, narrowing of coronary vessels may be involved in BPA-induced cardiotoxicity, increasing the risk of ischemic heart disease.

Like BPA, LP diet induced an inward remodeling of intramyocardial arteries. However, no additive effect of BPA and LP diet on this remodeling was observed. Although a previous study did not observe an effect of postweaning protein restriction on coronary flow (Murca et al., 2012), an increased wall/lumen ratio was found in the aorta of postweaning protein-restricted rats (Maia et al., 2014). Impaired nitric oxide bioavailability and oxidative stress could be mechanisms underlying vessel wall thickening in response to protein restriction (Maia et al., 2014) and to BPA (Ramadan et al., 2020). We cannot exclude that a longer and higher-dose BPA exposure would represent an additional risk factor for ischemic diseases in malnourished individuals.

Changes in heart morphology and function do not reflect the rise in blood pressure induced by short-term BPA exposure in LP diet-fed mice. Liver and lung water content were not affected by these two risk factors not indicating congestive heart disease. In addition, kidney mass, proteinuria, glomerular area, and expression of α-SMA were normal after LP diet combined or not with BPA exposure. Therefore, these data suggest no hypertensive glomerular damage following LP and BPA exposure. However, we found increased renal gene expression of the profibrotic factors in response to BPA exposure, particularly TGF-β1 in malnourished mice. Previous report have showed that maternal exposure to BPA affects kidney histomorphology in female but not in male mice (Nunez et al., 2018). Different mechanism underlying BPA-induced cardiovascular damage in malnourished males and females are possible and relevant but not addressed here.

Hypertensive effect of BPA exposure may be associated with altered autonomic nerves activity and/or expression of components of the renin-angiotensin system (RAS). Thoene et al. (2018) demonstrated an increase in the number of sympathetic fibers in the liver in response to 50 μg kg^−1^ day^−1^ BPA exposure, the same dose used in the present study. However, conflicting data have been reported for the autonomic control of heart rate and an increased and attenuated parasympathetic tone has been suggested (Pant et al., 2012; Belcher et al., 2015). More consistent, an upregulation of angiotensin II was reported in vascular tissue and cells exposed to BPA (Saura et al., 2014; Gao et al., 2019) and the antagonist of angiotensin II receptor type 1 (AT1) reversed the BPA-induced high blood pressure (Saura et al., 2014). Further studies assessing the concentration of angiotensin II or protein expression of the cardiac RAS components should be assessed. As the hypertensive effect of the postweaning protein restriction has also been related to sympathetic overactivity and RAS activation (Silva et al., 2015), we investigated if the combination of BPA and LP diet could have additional effect on tissue and circulating adrenergic catecholamines and RAS components. The plasma levels of catecholamines did not significantly change after LP diet or BPA exposure, although adrenaline levels were three times greater in BPA-exposed animals and may play a role on BPA adverse cardiovascular effect.

Genomic mechanisms have been associated to hypertensive and other adverse cardiovascular BPA effect (Wehbe et al., 2020; Zhang et al., 2020). Seq RNA analysis showed that aquatic exposure to low-dose BPA upregulates 22 RAS genes in rare minnow (Zhang et al., 2016). In the present study, short-term BPA exposure at presumed safety levels upregulated hepatic angiotensinogen gene in LP-fed mice. Cardiac AT1 and AT2 receptor gene expression was not affected. In classical RAS activation, angiotensinogen in the liver is substrate of renin released by the kidney to form angiotensin I, which is cleaved by ACE to form angiotensin II. Therefore, *Agt* upregulation could be an initial step for RAS activation in protein-restricted mice exposed to the BPA and a putative mechanism for the additional rise in blood pressure in this group. A summary of the altered cardiovascular parameters in response to protein restriction and/or BPA exposure found in the present study is shown in Supplementary Table 3.

In conclusion, the present study demonstrated that short-term exposure to low BPA concentrations increases blood pressure and induces diastolic dysfunction, cardiac fibrosis, and inward remodeling of coronary vessels. The hypertensive effect of BPA was even greater in protein-malnourished mice, associated with upregulated hepatic angiotensinogen gene. Therefore, adverse BPA cardiovascular effects may represent an additional cardiovascular risk following BPA exposure in malnourished population.

## Data Availability Statement

The original contributions presented in the study are included in the article/Supplementary Material, further inquiries can be directed to the corresponding author/s.

## Ethics Statement

The animal study was reviewed and approved by Research Animal Ethics Committee/UNICAMP.

## Author Contributions

MG-A, AD, AN, and EC designed the research. MG-A, EL-G, LC, TB, TA, LR, and MA performed the research. MG-A and AD prepared the manuscript. All the authors reviewed the manuscript.

## Conflict of Interest

The authors declare that the research was conducted in the absence of any commercial or financial relationships that could be construed as a potential conflict of interest.

## Supplementary Material

Supplementary Figure 1 Glomerular morphology and profibrotic gene expression in control and malnourished mice exposed to BPA. Representative images for H.E. **(A)**, quantification of glomerular area **(B)**, and α-smooth muscle actin (α-SMA) immunofluorescence images and quantification **(C,D)** of kidney sections of mice fed a normoprotein (control) or low-protein (LP) diet during 8 weeks and exposed to BPA for 9 days. CTGF **(E)** and TGF-β1 **(F)** mRNA expression was quantified in kidney samples. Data are expressed as the mean ± SEM (number of animals/group is indicated in the bars). One-way ANOVA followed by the Newman-Keuls test, ^*^*p* < 0.05 vs. control; ^&^*p* < 0.05 vs. LP. White bars in **(A)** and **(C)** represent 50 μm. G, glomeruli; a.u., arbitrary units.

Supplementary Table 1 Primer sequences.

Supplementary Table 2 Body and tissue weight and plasma catecholamine levels in mice fed a control or low-protein diet (LP) and exposed to bisphenol A (BPA). Data are expressed as the mean ± SEM; *n*, number of animals for each parameter. One-way ANOVA followed by the Newman-Keuls test, *p* < 0.05: ^*^ vs. Control; ^#^ vs. BPA. Adrenaline data were non-normally distributed and analyzed using the Kruskal-Wallis test (*P* > 0.05).

Supplementary Table 3 Summary of cardiovascular parameters alterations in response to protein restriction, BPA exposure or the combination of both. LVEDP, left ventricular end diastolic pressure. Highlighted arrows identify cardiovascular parameters that were only or more affected by the combination of protein restriction and BPA exposure. ↑, increase; =, no changes.
