# Supplementary figures and images for: Ventricular Fibrosis and Coronary Remodeling Following Short-Term Exposure of Healthy and Malnourished Mice to Bisphenol A
Source: Front Physiol. 2021 Apr 12;12:638506. doi: 10.3389/fphys.2021.638506 (PMC8072349; doi:10.3389/fphys.2021.638506)

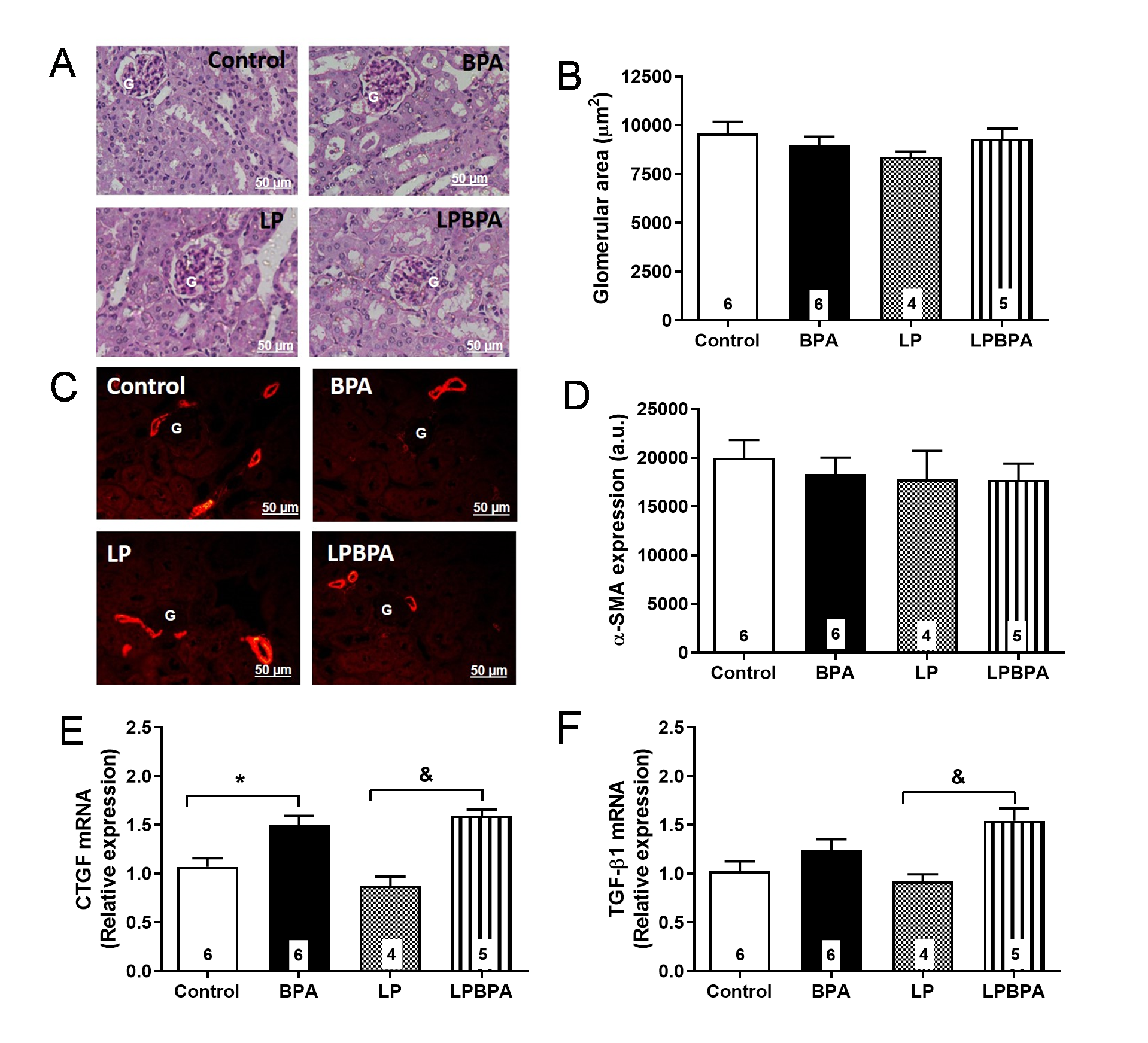

Supplement: Supplementary Figure 1 — Glomerular morphology and profibrotic gene expression in control and malnourished mice exposed to BPA. Representative images for H.E. (A), quantification of glomerular area (B), and α-smooth muscle actin (α-SMA) immunofluorescence images and quantification (C,D) of kidney sections of mice fed a normoprotein (control) or low-protein (LP) diet during 8 weeks and exposed to BPA for 9 days. CTGF (E) and TGF-β1 (F) mRNA expression was quantified in kidney samples. Data are expressed as the mean ± SEM (number of animals/group is indicated in the bars). One-way ANOVA followed by the Newman-Keuls test, *p < 0.05 vs. control; &p < 0.05 vs. LP. White bars in (A) and (C) represent 50 μm. G, glomeruli; a.u., arbitrary units. [file Image_1.TIF]
