# Supplementary material for: Ventricular Fibrosis and Coronary Remodeling Following Short-Term Exposure of Healthy and Malnourished Mice to Bisphenol A
Source: Front Physiol. 2021 Apr 12;12:638506. doi: 10.3389/fphys.2021.638506 (PMC8072349; doi:10.3389/fphys.2021.638506)
Supplement: Supplementary Table 1 — Primer sequences. [file Table_1.DOCX]

| Gene | Forward (5´-3´) | Reverse (5´-3´) | NM |
| --- | --- | --- | --- |
| *Agtr1a* | AGTTGGGAGGGACTGGATGA | GTTAAGTCCGGGAGAGCAGC | 177322 |
| *Agtr1b* | CACTGTAGATGGGGAGCAGCCAA | GAGAGTAGGGATCATGACAA | 175086 |
| *Agtr2* | GCTTACTTCAGCCTGCATTT | GGACTCATTGGTGCCAGTTG | 007429 |
| *Agt* | CGGAACGACCTCCTGACTTG | ATTGAGAACCTCTCCCACTCG | 007428 |
| *Ctgf* | CACAGAGTGGAGCGCCTGTTC | GATGCACTTTTTGCCCTTCTTAATG | 001139999.1 |
| *Tfgβ1* | CCAAGGAGACGGAATACAGG | TTTGGGGCTGATCCCGTTG | 011577.2 |
| *36B4* | GAGGAATCAGATGAGGATATGGGA | AAGCAGGCTGACTTGGTTGC | 007475.5 |

**Supplemental Table 1.** Primer sequences.

|  | **Control** | **BPA** | **LP** | **LPBPA** | **(n)** |
| --- | --- | --- | --- | --- | --- |
| Initial body  weight (g) | 16.3 ± 3.2 | 17.0 ± 2.6 | 15.9 ± 2.5 | 15.9 ± 2.6 | (13) |
| Final body  weight (g) | 38.9 ± 1.4 | 39.2 ± 0.8 | 31.7 ± 2.6 * | 31.3 ± 2.1 ^#^ | (9-12) |
| Heart weight (g) | 0.19 ± 0.01 | 0.18 ± 0.01 | 0.12 ± 0.01 ***** | 0.13 ±0.01 ^#^ | (9-12) |
| Heart weight/  body weight (g/g) | 0.50 ± 0.04 | 0.48 ± 0.03 | 0.41 ± 0.02 | 0.45 ± 0.03 | (9-12) |
| Kidney weight (g) | 0.36±0.03 | 0.39±0.04 | 0.30±0.01 | 0.30±0.02 | (9-12) |
| Kidney weight/body weight (g/g) | 0.64±0.02 | 0.64±0.02 | 0.66±0.02 | 0.65±0.03 | (9-12) |
| Lung weight (g) | 0.38±0.05 | 0.31±0.04 | 0.47±0.02 | 0.39±0.04 | (4-6) |
| Lung weight/body weight (g/g) | 0.90±0.001 | 0.75±0.001 | 1.15±0.001 | 1.03±0.001 | (4-6) |
| % water lung | 4.74±0.11 | 4.48±0.1 | 5.03±0.17 | 4.72±0.07 | (4-6) |
| Liver weight/body weight (g/g) | 3.97±0.38 | 4.37±0.21 | 4.63±0.18 | 4.65±0.26 | (4-6) |
| % water liver | 2.99±0.11 | 3.2±0.06 | 3.09±0.09 | 3.22±0.07 | (4-6) |
| Noradrenaline (ng/mL) | 7.69±1.61 | 8.98±1.07 | 6.50±1.81 | 10.22±0.96 | (4-6) |
| Adrenaline  (ng/mL) | 4.63±3.00 | 14.89±6.01 | 4.51±1.84 | 17.09±8.29 | (4-6) |

**SupplementalTable 2.** Body and tissue weight and plasma catecholamine levels in mice fed a control or low-protein diet (LP) and exposed to bisphenol A (BPA).

Data are expressed as the mean ± SEM; n= number of animals for each parameter. One-way ANOVA followed by the Newman-Keuls test, p<0.05: * *vs*. Control; ^#^ *vs*. BPA. Adrenaline data were non-normally distributed and analyzed using the Kruskal-Wallis test (P>0.05).

**Supplemental Table 3**. Summary of cardiovascular parameters alterations in response to protein restriction, BPA exposure or the combination of both.

|  | Protein restriction | BPA exposure | Combination |
| --- | --- | --- | --- |
| Blood pressure | **↑** | **↑** | **↑↑** |
| LVEDP | **=** | **↑** | **↑** |
| Ventricular fibrosis | **=** | **↑** | **↑** |
| Coronary remodeling | **↑** | **↑** | **↑** |
| Plasma adrenaline | **=** | **↑** | **↑** |
| Angiotensinogen gene expression | **=** | **=** | **↑** |
| Cardiac CTGF mRNA expression | **=** | **↑** | **↑** |
| Cardiac TGF-β1 mRNA expression | **=** | **=** | **↑** |

LVEDP: left ventricular end diastolic pressure. Highlighted arrows identify cardiovascular parameters that were only or more affected by the combination of protein restriction and BPA exposure. ↑: increase; =: no changes.
